# The complete chloroplast genome sequence of *Erigeron breviscapus* and *Erigeron multiradiatus* (Asteraceae)

**DOI:** 10.1080/23802359.2019.1683478

**Published:** 2019-10-30

**Authors:** Zhong-Ji Li, Ying-Ying Liu, Cong-Wei Yang, Zi-Gang Qian, Guo-Dong Li

**Affiliations:** a Faculty of Traditional Chinese Pharmacy, Yunnan University of Chinese Medicine, Kunming, China;; b Yunnan Insitute for Food and Drug, Kunming, China;; c Yunnan Key Laboratory for Dai and Yi Medicines, Yunnan University of Chinese Medicine, Kunming, China

**Keywords:** *Erigeron breviscapus*, *Erigeron multiradiatus*, complete chloroplast genome, Asteraceae

## Abstract

The complete chloroplast genomes of *Erigeron breviscapus* (Vant.) Hand-Mazz and *Erigeron multiradiatus* (Lindl.) Benth were reported in this study. The complete chloroplast genomes were 152,367 bp and 152,281 bp for *E. breviscapus* and *E. multiradiatus*, respectively. *Erigeron breviscapus* including two inverted repeat (IRs, 24,692 bp) regions, one large singe copy region (LSC) and one small singe copy region (SSC) of 84,881 bp and 18,102 bp, whereas *E. multiradiatus* contained IRs of 24,691 bp, LSC of 84,789 bp and SSC of 18,110 bp. The chloroplast genomes both contained 129 genes, including 84 protein-coding genes, 37 tRNA genes, and 8 rRNA genes.

*Erigeron breviscapus* (Vant.) Hand-Mazz, recorded in Chinese Pharmacopeia, was one of the most important and most recognized species of Erigeron genus (Chinese Pharmacopoeia Commission, 2015). Scutellarin is an important bioactive ingredient in *E. breviscapus* (Qu et al. 2001; Yang et al. 2008). It was also one of the major flavonoid constituents in *Erigeron multiradiatus* (Lindl.) Benth, belonging to the same genus as *E. breviscapus* (Zhang et al. 2008). As an economically important medical herb, *E. breviscapus* is increasingly endangered due to overexploitation and shrinkage of its habitats. However, most previous researches were engaged in its medicinal properties, and only a few studies have been carried out on its conservation genetics and population diversity (Li et al. 2013). Here, we sequenced the cp genomes for *E. breviscapus* and *E. multiradiatus* and analyzed the genome features, which will provide useful information for the further study of the genus.

Two voucher specimens were deposited in the Herbarium of Yunnan University of Chinese Medicine (YNUTCM), China, including *E. breviscapus* (voucher ML003-20180705) from the Mile, Yunnan Province, China (N24°46′, E103°26′) and *E. multiradiatus* (voucher MEK001-20180822) from the Maerkang, Sichuan Province, China (N32°00′, E102°32′). Total genomic DNA was extracted using plant DNA (Bioteke Corporation, China). A library was constructed and sequencing was performed on an Illumina HiSeq 2500 platform (Illumina Inc., SanDiego, CA). A total of 7 Gb reads were obtained and de novo assembled using NOVOPlasty (Dierckxsens et al. 2017). The complete cp genome was annotated with the online annotation tool GeSeq (Tillich et al. 2017). All of the plastomes were aligned using MAFFT v.7 (Katoh and Standley 2013), and the RAxML (Stamatakis 2014) inference was performed by using GTR model with support for branches evaluated by 1000 bootstrap replicates.

The complete chloroplast genome of *E. breviscapus* (MN449489) was 152,367 bp in length, including two inverted repeat (IRs, 24,692 bp) regions, one large singe copy region (LSC) and one small singe copy region (SSC) of 84,881 bp and 18,102 bp, the overall GC content of the whole genome is 37.1%. On the other hand, the complete chloroplast genome of *E. multiradiatus* (MN449490) was 152,281 bp in length. Separating LSC of 84,789 bp and SSC of 18,110 bp, a pair of IRs was 24,691 bp, the overall GC content is 37.2%. The chloroplast genomes both contained 129 genes, including 84 protein-coding genes, 37 tRNA genes, and 8 rRNA genes.

Phylogenetic analysis was conducted based on 17 published chloroplast genomes to infer phylogenetic position of *E. breviscapus* and *E. multiradiatus* within the family of Asteraceae (Figure 1). The result strongly supported *E. multiradiatus* and its congeneric species, *E. breviscapus*, as sister group with 100% bootstrap value. The data will provide a useful resource for studying the genetic diversity of *E. multiradiatus* and *E. breviscapus*, and the phylogenetic relationships of the Asteraceae family.

**Figure 1.**
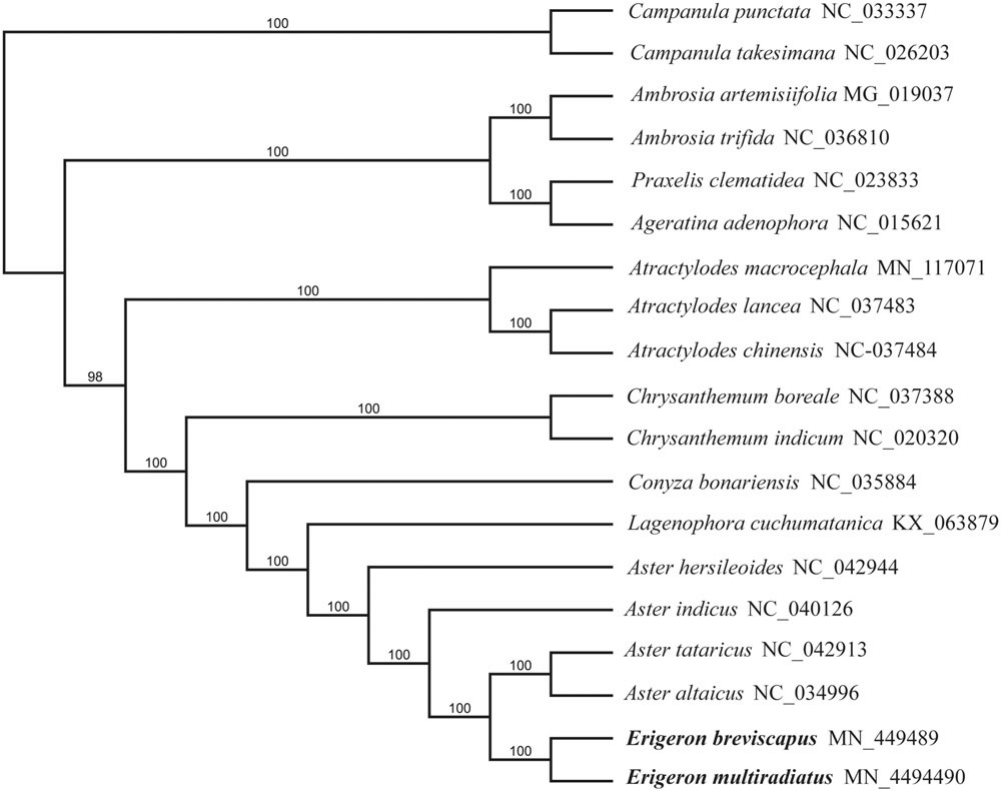
The Maximum likelihood tree of *E. breviscapus* and *E. multiradiatus* and other Asteraceae species based on whole chloroplast genome sequence, with Campanulaceae as outgroup. Bootstrap support values (based on 1000 replicates) are shown next to the nodes.
